# Low Level Laser Therapy to Reduce Recurrent Oral Ulcers in Behçet's Disease

**DOI:** 10.1155/2016/4283986

**Published:** 2016-07-31

**Authors:** D. B. Gandhi Babu, Sunanda Chavva, Shefali Waghray, Neeharika Satya Jyothi Allam, Marella Kondaiah

**Affiliations:** Department of Oral Medicine and Radiology, Panineeya Mahavidyalaya Institute of Dental Sciences and Research Centre, Hyderabad 500060, India

## Abstract

Behçet's disease (BD) is a chronic, relapsing multisystemic vascular condition. Behçet's disease was described by Hulusi Behçet in 1937. This rare multisystem relapsing-remitting inflammatory disease is poorly understood but is thought to be an autoimmune inflammatory vasculitic process in a genetically predisposed population. Diagnosis of Behçet's disease is based on International Criteria of Behçet's Disease (ICBD). The present paper describes a case report of Behçet's syndrome where aphthous stomatitis was treated with low level laser therapy.

## 1. Case Report

A 17-year-old male patient came to the department of oral medicine with a chief complaint of painful ulcers in the mouth since 3 years. There was history of h/o of exacerbation of ulcers since 4 days. These ulcers were recurrent and multiple posing difficulty in eating and speech. Alongside, history of ulcers on trunk, scrotum, and lower extremities since three years was given. Initially ulcers appeared in the mouth; later they developed on trunk, genital region, and lower extremities. Patient also gave history of anterior uveitis.

Patient visited several doctors for the same problem but did not get permanent relief. Recent medical reports revealed an increased white blood cell count (14600/cumm), neutrophil count 82%, and ESR (1st hr 70 mm, 2nd hr 92 mm). Patient was earlier treated with doxycycline 100 mg twice for 5 days, analgesic, mupirocin 2% skin ointment, and triamcinolone acetonide 0.1% buccal paste. But lesions did not show much remission.

**Figure 1:**
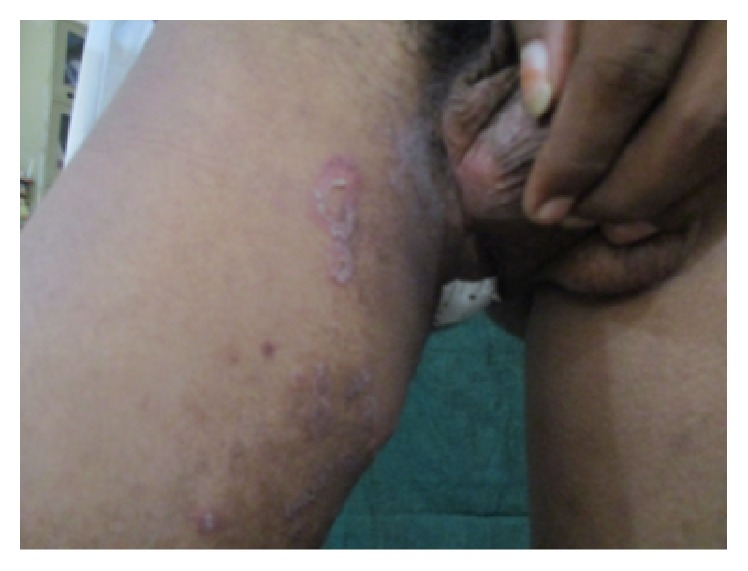
Ulcers seen on the scrotum.

**Figure 2:**
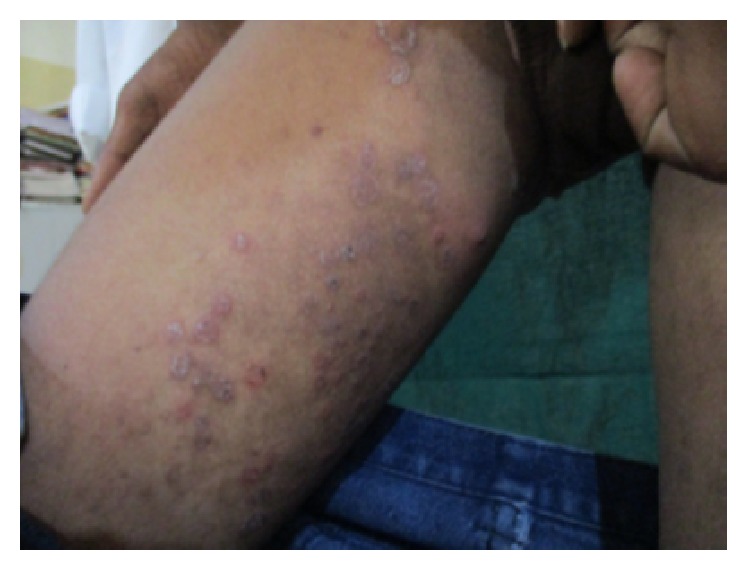
Vasculitic lesions on the legs.

On general examination there were ulcers on scrotum (Figure 1) and vasculitic lesions on legs (Figure 2) and trunk. Temporomandibular joint (TMJ) and other joints examination revealed no abnormality. Pallor and lymphadenopathy were positive. Two discrete lymph nodes in relation to right and left submandibular region were palpable which are of size 1 cm each, soft, tender, and not fixed to underlying structures.

**Figure 3:**
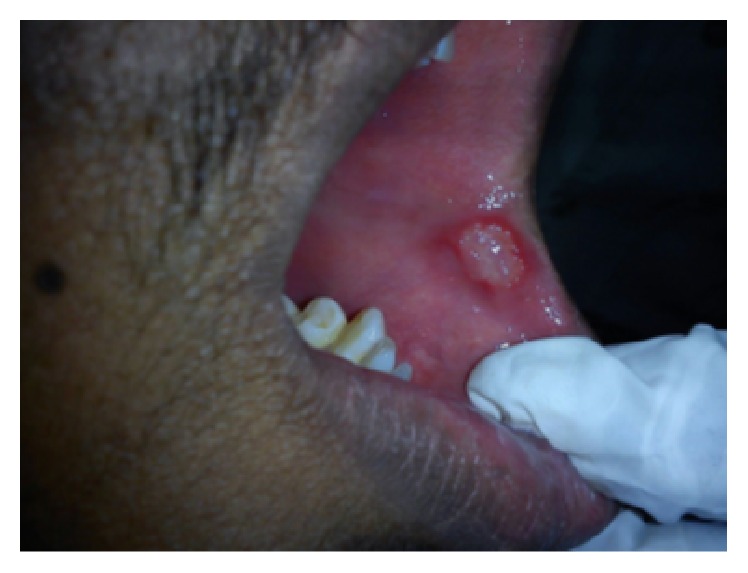
Ulcers on left commissure of buccal mucosa.

**Figure 4:**
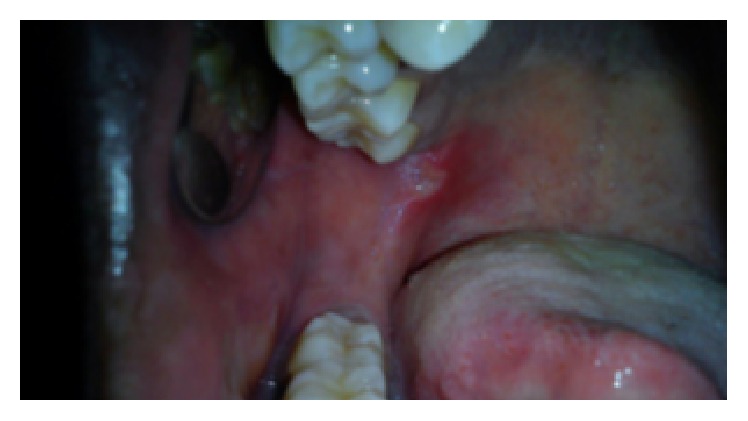
Ulcer at the retromolar region of the mouth.

On intraoral examination, there were discrete multiple ulcers seen on left commissure of buccal mucosa (Figure 3), retromolar region (Figure 4), and superomedial to right retromolar area. The ulcers were ovoid in shape, symmetrical, and shallow, with size varying from 5 mm to 1 cm with sloping margins. Ulcers were surrounded by erythematous halo. Floor of the ulcer was covered with pseudomembranous slough. On palpation, all inspectory findings were confirmed. The ulcers were tender with no induration at borders and margins. Pathergy test was negative (Figure 7). Based on the history and clinical presentation of the lesions which fulfilled Behçet's Criteria, a provisional diagnosis of Behçet's disease was given.

Patient was treated with triamcinolone acetonide 0.1% ointment and 100 mg doxycycline one/day for one week. Patient was reviewed after a week, and the lesions showed remission. On the follow-up visit of 15 days, new lesions were seen on the right buccal mucosa and lower labial mucosa. Patient was advised to continue topical triamcinolone acetonide for two weeks. On the third visit, new lesions were seen on the tongue and also the previous lesions persisted with burning sensation of mouth.

**Figure 5:**
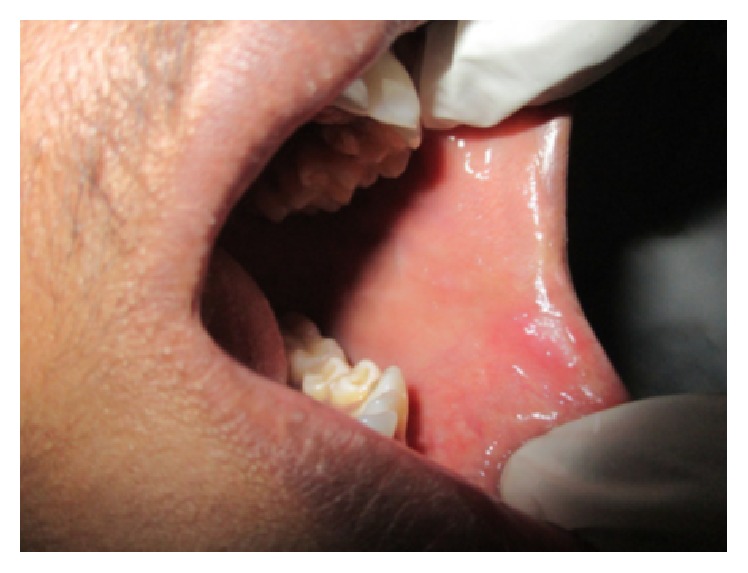
Healed ulcers on left commissure of buccal mucosa.

**Figure 6:**
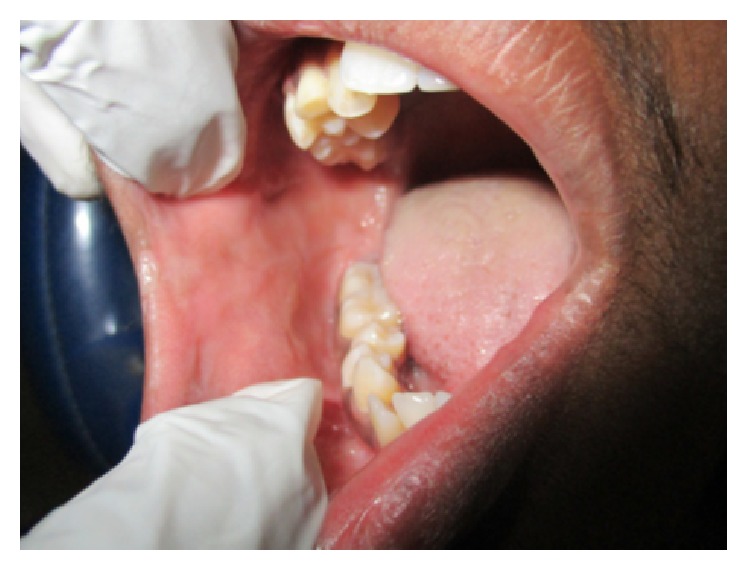
Healed ulcers on retromolar region of mouth.

**Figure 7:**
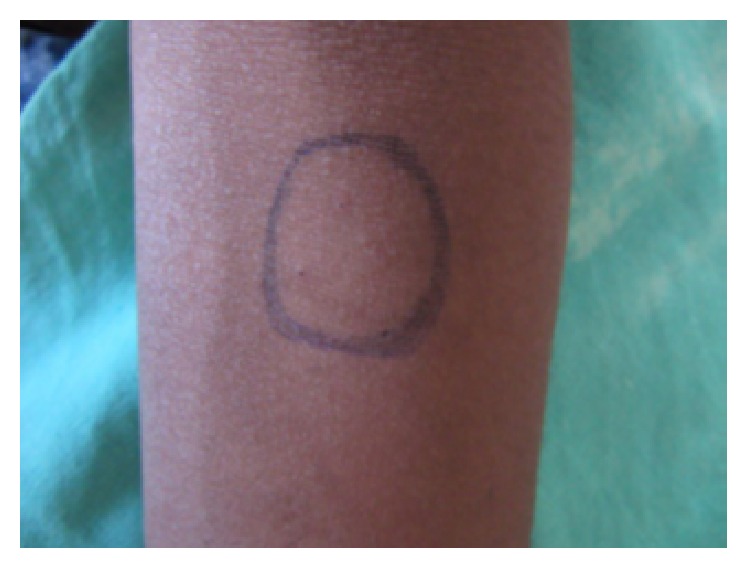
Negative pathergy test.

Hence photobiostimulation with low level laser therapy was planned. 980 nm wavelength diode laser was used in noncontact mode with 5-watt energy, in pulsed mode for 45 seconds per session. Two such sessions were performed. Patient showed spontaneous relief of pain and burning sensation where VAS showed significant improvement from 9 to 1 and all the lesions showed remission within 4-5 days and showed no recurrence since seven months (Figures 5 and 6).

## 2. Discussion

Behçet's disease (BD) was initially described by Turkish dermatologist Hulusi Behçet in 1937 [1]. BD is systemic vasculitis characterized by hyperactivity of neutrophils with enhanced chemotaxis and elevated proinflammatory cytokines. The HLA-B51 genotype is frequently linked to most BD. It generally occurs within the age range of 25–45 years [2]. A distinct geographical variation was noted in gender distribution in BD; studies from Middle East and Turkey showed definite male predominance while in European countries there was nearly equal male and female distribution [3]. The present case was 17-year-old male. Cases of Behçet's disease seem to cluster along the ancient Silk Road, around Eastern Asia and Mediterranean basin [4]. Hence Behçet's disease is also called Silk Road Disease.

Diagnosis of Behçet's disease uses various classification systems. However, International Criteria for Behçet's Disease (ICBD) is used frequently. ICBD use six items: oral aphthae, genital aphthae, skin lesions, eye lesions, and positive pathergy test. In the ICBD, oral and genital ulcers and eye lesions are given 2 points each and the rest get one point each. Three or more points are needed to be diagnosed as BD [5, 6]. The present case presented with oral, genital, skin, and eye involvement and was classified as BD.

The mucocutaneous lesions are the characteristic features of BD with oral mucosa as the most common site [4]. Eye lesions include uveitis, retinal vasculitis, vascular occlusion, optic atrophy, conjunctivitis, and ultimately blindness. Skin lesions resembling erythema nodosum or large pustular lesions occur in over 50% of patients. The present case had lesions on oral mucosa, scrotum, inner part of thigh, and trunk of the body along with burning of eyes.

Although less common, in few, central nervous system (CNS) involvement is seen [7]. Involvement of large vessels is life threatening because of the risk of arterial occlusion [8]. Arthritis can occur in greater than 40% of patients. Most of the times, there is asymmetrical involvement with recurrence which rarely causes permanent joint damage [8]. The present case did not exhibit any symptoms of CNS involvement and arthritis.

Positive pathergy test is one amongst the criteria of Behçet's disease. The mechanism underlying pathergy is unknown. It is suggested that, on skin biopsy, an increased and abnormal cytokine release from keratinocytes in epidermis/dermis results in a perivascular infiltration. However the pathergy phenomenon is not constant during the course of the disease. The degree of positivity may occasionally correlate with disease activity [9]. The present case did not show a positive pathergy phenomenon.

Management of Behçet's disease is guided by organ involvement. Topical preparations of local anaesthetics and steroids provide symptomatic relief and are usually effective for mucocutaneous involvement [10]. Yurdakul et al. found that colchicine decreased the occurrence of genital ulcers, arthritis, and erythema nodosum in men and women [11]. De Souza et al. in a randomised control trail showed that colchicine is an effective treatment for mucocutaneous and joint manifestations in BD [12]. Alternative treatment for mucocutaneous involvement is pentoxifylline, dapsone, and cyclosporine and in refractory cases thalidomide is used [10].

Photobiostimulation by low level laser therapy has also been tried and studies showed its efficacy in treating aphthous stomatitis. Laser therapy is known to aid in tissue repair through enhancing its regenerative potential, microcirculation, cell metabolism, and neovascularisation. Lasers are known for their anti-inflammatory and analgesic action, with effect on lymphocyte metabolism and prostaglandin (PGE2) production [13, 14]. Studies done by Davatchi et al. [13] and Marinho and Giovani [14] and meta-analysis by Albrektson et al. [15] showed that low level laser therapy is an effective treatment modality in treating aphthous ulcers in patients with BD.

In a randomised control trial by Albrektson et al., effect of low level laser therapy on RAS was studied and was found to be effective [15]. In another meta-analysis by Enwemeka et al., low-power lasers of wavelength < 500 mW were effective in pain control and tissue repair of ulcers [16]. The present case also showed significant improvement in terms of burning sensation and recurrence of the lesion.

## 3. Conclusion

Behçet's disease is a multisystemic vascular disorder, with recurrent attacks and multisystem involvement. With recurrent lesions, quality of life is severely affected in patients. Management of Behçet's disease is mostly symptomatic and is targeted to the specific organ systems involved. However, there is no definitive treatment for Behçet's disease. Photobiostimulation with low level laser therapy employed in the present case not only showed instant pain relief but also reduced the recurrence period of the lesion. As topical and systemic medications such as steroids are avoided, risk of overdose and side effects can be prevented. Meticulous use of lasers can help in providing safe and clinically effective treatment to the aphthous stomatitis in Behçet's disease.
